# GHRH-antagonists in TNBC

**DOI:** 10.18632/oncotarget.3271

**Published:** 2015-01-05

**Authors:** Jörg B. Engel

**Affiliations:** Department of OB/Gyn, Head of Gynecologic Oncology, Medical University of Gießen, Gießen, Germany

“Triple negative breast cancer” (TNBC) represents a subgroup of breast cancers (BC), which do not express receptors for either estrogen or progesterone and which do not overexpress HER-2 receptors. Although this subgroup is characterized by immunohistochemistry, there is a seventy percent overlap with basal-like breast cancers, defined by gene-expression profiling and thus these terms are frequently used as synonyms. Because of this lack of therapeutic targets, TNBC is burdened with a dismal prognosis as compared to other subgroups of breast cancer and thus, a taxane- and anthracycline-based chemotherapy are the only options for patients with TNBC [1].

Various clinical studies investigated the effect of neoadjuvant chemotherapy in TNBC, i.e. systemic treatment was administered prior to surgery (as a means of an in vivo sensitivity testing). Two subgroups of TNBC with a distinct prognosis can be distinguished, based on the response to this neoadjuvant chemotherapy. Tumors, which are sensitive to chemotherapy as mirrored by a pathologic complete response (PCR), i.e. no surviving tumor tissue is detected in the surgical specimen, and cancers which are chemoresistant, as reflected by residual disease being detected after neodjuvant chemotherapy. While the former patient group has a good prognosis, overall survival of patients with chemoresistant TNBC is dramatically decreased [2]. These clinical observations emphasize, that it is of paramount importance to identify ways to overcome the chemoresistance of TNBC.

GHRH antagonists inhibit the growth of various experimental human cancers [3] including TNBC [4]. These peptide hormone analogs suppress tumor growth through both direct and indirect pathways. It was observed that GHRH-antagonists can inhibit the proliferation of diverse cancer lines by direct action *in vitro*, under conditions in which the contribution of the hypothalamic GHRH/pituitary GH/hepatic IGF-I axis is clearly excluded [5]. These studies led to the conclusion that the main mechanism responsible for tumor inhibition could be a direct effect of the antagonists on the tumor tissue. In more than 70 % of TNBC, mRNA for receptors for GHRH and its respective ligands was detected and accordingly tumor growth of TNBC cell lines could be powerfully decreased by antagonists of GHRH [4]. The indirect, endocrine mechanism operates through the suppression of the release of GH from the pituitary, and the resulting inhibition of the hepatic production of IGF-I [3].

The current publication by Perez et al. [6] further extends our knowledge on the therapeutic potential of GHRH antagonists. This experimental study was carried out by the group of Nobel laureate Dr. A.V. Schally, the leading world expert on agonistic an antagonistic analogs of GHRH. The current study demonstrates synergy of GHRH-antagonist MIA-602 with doxorubicin as shown in an *in vivo* model of TNBC. More importantly, the authors clearly document *in vivo* that the resistance to doxorubicin of human MX-1 TNBC can be overcome by the addition of treatment with MIA-602. Thus, treatment of doxorubicin-resistant MX-1 TNBC with MIA-602 can reactivate sensitivity to doxorubicin mirrored by the synergistic effect of combination treatment; doxorubicin administered as single agent has no effect on this tumor's growth. A possible explanation for this important finding, is the suppression of the expression of the multi-drug resistance factors, MDR-1 and NANOG, by MIA-602, which has been demonstrated *in vivo*. In addition, in order to demonstrate that the decrease of this expression is mechanistically relevant, it was shown that treatment with MIA-602 inhibits the function of the efflux pump coded by the MDR-1 gene. Thus, strong evidence is presented, that besides being a potent tumor inhibiting agent, which shows a synergistic effect with anthracycline treatment, GHRH antagonist MIA-602 is also an inhibitor of the MDR-1 drug efflux pump. Accordingly MIA-602 can overcome resistance to doxorubicin in cancer therapy. The results presented are clinically relevant as resistance to anthracycline is a major problem in the treatment of TNBC, The prognosis of chemoresistant TNBC is thus significantly decreased. Accordingly, we believe that phase I studies should be carried out investigating combined treatment of MIA-602 and doxorubicin in patients with locally advanced or metastatic anthracycline resistant TNBC.

## References

[1] Yadav BS (2014). World J Clin Oncol.

[2] von Minckwitz G (2012). Ann Oncol.

[3] Schally AV (2008). Nat Clin Pract Endocrinol Metab.

[4] Köster F (2009). Breast Cancer Res Treat.

[5] Kiaris H (2003). Expert Opin Investig Drugs.

[6] Perez R (2014). Oncoscience.

